# Recent Achievement in Gene Cloning and Functional Genomics in Soybean

**DOI:** 10.1155/2013/281367

**Published:** 2013-11-07

**Authors:** Zhengjun Xia, Hong Zhai, Shixiang Lü, Hongyan Wu, Yupeng Zhang

**Affiliations:** ^1^Key Laboratory of Soybean Molecular Design Breeding, Northeast Institute of Geography and Agroecology, Chinese Academy of Sciences, Harbin 150081, China; ^2^Jiangsu Academy of Agricultural Sciences, Nanjing 210014, China

## Abstract

Soybean is a model plant for photoperiodism as well as for symbiotic nitrogen fixation. However, a rather low efficiency in soybean transformation hampers functional analysis of genes isolated from soybean. In comparison, rapid development and progress in flowering time and photoperiodic response have been achieved in *Arabidopsis* and rice. As the soybean genomic information has been released since 2008, gene cloning and functional genomic studies have been revived as indicated by successfully characterizing genes involved in maturity and nematode resistance. Here, we review some major achievements in the cloning of some important genes and some specific features at genetic or genomic levels revealed by the analysis of functional genomics of soybean.

## 1. Introduction

Soybean (*Glycine max* (L.) Merr.) is an important crop that provides a well-balanced source of protein and oil. In addition, most of the components of soybean such as *α*-linolenic acid and isoflavones have beneficial health effects. Recently, genomic studies have given more evidence that domestication of soybean began as early as five thousand years ago in China [1], although multiple origins of soybean domestication in the Eastern Asian region including China, the Korean peninsula, and Japan were proposed [2]. Soybean is a wonderful model plant for photoperiodism study [3, 4]. In the 1920s, studies on the relationship between daylength and flowering time in soybean, tobacco, and other plants led to the discovery of photoperiodism. Also soybean is the only staple crop that is capable of fixing atmospheric nitrogen through symbioses with soil-borne microorganisms. However, as *Arabidopsis* [5] and rice [6] are becoming more popular model plants, soybean studies are somewhat lagging behind in terms of papers published with high impact factor per year possibly due to the lower efficiency in transformation and genome complexity. As sequence information of the reference genome of soybean cultivar, Williams 82, has been available since 2008 and formally published in Nature in 2010 [7], a new era of gene cloning and functional analysis in soybean is emerging.

## 2. Positional Cloning and Functional Analysis of Genes Controlling Flowering and Maturity

Flowering is one of the most important ecological and agronomical traits since it is related to the domestication, latitudinal and ecological adaption, and yield directly. About ten major quantitative trait loci (QTLs) for flowering time have been reported in soybean [8–15]. The interactions between major QTLs have been studied intensively among different environments and geographical locations. The *E* serials (*E1* to *E8*) are controlling flowering time, duration of the reproductive phase (DRP) [16], and other physiological or agronomical traits, such as branching [17], yield [18], and chilling resistance [19, 20]. Many researchers were involved in the identification of molecular basis for *E* locus in soybean [21, 22]. In particular, cooperative researches from Japan and China have cloned *E1*, *E2*, *E3*, and *E4* genes. In 1998, *E4* gene was identified to encode phytochrome A2 protein, by the candidate gene approach based on the QTL position on the map [23]. In the following year, the *E3* gene was successfully cloned by positional cloning using residual heterozygous line (RHL) [24]. Both *E3* and *E4* are involved in response to the light quality (red to far-red quantum (R : FR) ratios); however, their function pathways are different but overlapping [25, 26]. In addition, *E3* gene has a dominant effect over *E4* gene since *E4* genotype only showed its own phenotype under *E3* genetic background. The cloning result showed that molecular basis of *E3* gene is a copy of the phytochrome, *GmPhyA3*. In soybean, there is a third *GmphyA* gene, *GmPhyA1*, whose function needs further characterization [23]. Molecular basis for *E2* locus was identified with the same strategy as the one used in cloning of *E3* [27]. *GmGIa* (*Glyma10g36600*) has been proven to be the genetic factor underlying the *E2* locus [27]. The *GIGANTEA* (*GI*) gene in *Arabidopsis* has been proven to play an important role in GI-CO-FT mediated photoperiodic flowering. However, in soybean, the flowering time phenotype difference between dominant and recessive alleles of *E2* appeared to be independent of daylength, inferring that this locus is not significantly associated with photoperiodic response.

The *E1* locus was genetically identified in 1971 [8], possibly the same locus as *E* or *S* locus having a major genetic effect on controlling flowering time, which was already perceived in the 1920s when people discovered the photoperiodism [8, 28, 29]. Although many researchers have tried to decipher the molecular basis *E1* locus in soybean [21, 30], it ended with a plausible guess or a closed genetic distance since this gene is located in the pericentromeric region with low recombination rate [7, 31].

Successful identification of the molecular basis of the soybean maturity locus *E1* will help us to understand the regulation of flowering time and maturity in soybean. After nearly ten-year effort, *E1* was proven to be a legume-specific gene having a putative bipartite nuclear localization signal (NLS) coupled with a domain distantly related to B3 [31]. The suppressed expression in short days is very much consistent with the notion that *E1* is a flowering repressor and under photoperiodic regulation.

The flowering promoting factors called florigen are transported from leaves to the shoot or lateral apical meristems through the phloem in a regulated manner to provoke the initiation of floral meristems [32]. The protein encoded by *FLOWERING LOCUS T* (*FT*) in *Arabidopsis* [33] and its ortholog in rice [34] were first proven to be part of the long-sought florigen. FTs are largely conserved among different plant species; however, the regulation of FT is quite diversified from species to species [35]. Many genetic factors are controlling photoperiodic flowering in soybean through two homologs (*GmFT2a* and *GmFT5a*) of *FLOWERING LOCUS T *(*FT*) to provoke the initiation of floral meristems [36]. For the stem termination, also known as growth habit, the main function gene is *GmTFL1b *[37, 38].

Although four major genes, *E1* to *E4* along with *GmFT2a/5a* and *DT1*, have been cloned, the flowering gene network is almost unknown. In addition, there are a vast number of *Arabidopsis* flowering genes in the genome of soybean [39]. Further characterization of these sequences will shed light on our deep understanding of gene specification, diversification, and evolution of flowering genes during domestication and natural evolution.

## 3. Positional Cloning of Resistance Genes to Biotic and Abiotic Stresses

Soybean cyst nematode (*Heterodera glycines* Ichinohe) is a major constraint to soybean production worldwide. This nematode disease causes more than US$1 billion in yield losses annually in the United States [40]. Recently, two important genes, *Rhg4* and *Rhg1,* have been cloned and functionally characterized. *Rhg4* (for resistance to *Heterodera glycines 4*) locus is a major quantitative trait locus contributing to resistance to this pathogen. Positional cloning reveals that the corresponding gene encodes a serine hydroxymethyltransferase, an enzyme (SHMT) that is ubiquitous in nature and structurally conserved across plant kingdoms. The enzyme functions as interconversion of serine and glycine, involved in cellular one-carbon metabolism [40]. Various function methods, such as mutation analysis, gene silencing, and transgenic complementation, all confirmed that this gene confers resistance. On the other hand, most SCN-resistant soybeans in the Midwest, USA, are bred to contain *Rhg1* (*rhg1-b*). After positional cloning and* Rhg2-b* gene silencing, genes in a 31-kilobase segment at *rhg1-b* encode three types of functional proteins, an amino acid transporter, an *α*-SNAP protein, and a WI12 (wound-inducible domain) protein, each contributes to resistance [41]. Ten tandem copies are present in an *rhg1-b* haplotype; in comparison, only one copy of the 31-kilobase segment per haploid genome in susceptible varieties is existing. Overexpression of individual genes in roots is not sufficient; only overexpression of these genes together can gain enhanced SCN resistance. This result showed an interesting new insight into our understanding of disease resistance that copy number variation increases the expression of a set of dissimilar genes in a repeated multigene segment [41].

Soybean cultivars carrying *Rps1-k* locus are resistant to most races of *Phytophthora sojae* [42–45]. Five corresponding *Rps* genes, including the important *Rps1-k*, have been successfully mapped to the *Rps1* locus, on molecular LGN of soybean genetic map. Two classes of functional coiled coil-nucleotide-binding leucine-rich repeat (CC-NB-LRR)-type resistance genes, which belong to the larger NBS-LRR resistance gene family, are confirmed to confer race-specific *Phytophthora* resistance through positional cloning strategy [42].

*Rag1*, dominantly conferring resistance to the soybean aphid (*Aphis glycines *Matsumura), was previously mapped from the cultivar Dowling to a 12 cM interval on soybean chromosome 7 (LG M). Kim et al. (2010) carried out further fine mapping and successfully delimited the region to 115 kb [46].

For abiotic stress, a QTL conferring Cl^−^ accumulation in the aerial part of soybean was named in 1969 by Abel [47]. This locus was confirmed by Lee et al. in 2004 using different genetic materials [48]. Recently, a major salt-tolerant QTL was also mapped to LG N, putatively the same position [49]. However, whether the salt resistant gene commonly exists between wild and cultivated soybean still needs to be confirmed. Tuyen and his team have reported a new QTL for alkaline salt tolerance and the candidate region has been narrowed using RHL line. Although the functional gene has not deciphered, the adjacent markers can be used for MAS to pyramid tolerance genes [50]. Funatsuki et al. (2005) reported chilling-tolerant QTLs—the qCTTSW 1, 2, and 3 QTLs [19], and Ikeda et al. (2009) identified a new one tightly linked to Sat_162 on LG A2 and specifically involved in controlling seed development at low temperature [51].

Soybean is considered to be one of the most drought sensitive crops, with approximately 40% reduction of the yield in the worst years [52]. As a consequence of global warming, the drought stress will become more serious than ever before. Several researchers have mapped QTLs for drought [52] or its related trait, for example, canopy wilting [53]. Due to the complexity of this trait, unwinding of the molecular basis is still a big challenge. Other researchers identified waterlogging tolerance (WLT) [54]. 

The availability of physical and genetic maps of soybean and other legume will accelerate the cloning and functional confirmation of QTL genes conferring various agronomic traits. Over 100 traits have been mapped in the last 18 years. Current status of QTL mapping along with the other soybean genomic information can be found at SoyBase (http://soybase.org/) [55].

## 4. Other Important Traits Related to Agronomic Traits

*Arabidopsis* JAGGED (JAG) homolog in soybean, designated as Gm-JAGGED1, has been proven to have pleiotropic effect on narrow leaflet and fruit patterning [56]. Positional cloning has narrowed down QTL region to a single gene level for both traits [56, 57]. Both single trait controlled by many QTL genes and individual gene having multiple pleiotropic effect make soybean genome intriguing; care needs to be taken when explaining the result of functional analysis of soybean genes.

Sayama et al. (2012) revealed that a single locus, *Sg-1* encoding a UDP-sugar-dependent glycosyltransferase (*Glyma07g38460*), is responsible for the structural diversity of glycosylation of triterpenoid saponins of soybean [58]. 

## 5. Specific Features for Soybean Genome

Du et al. (2012) revealed biased accumulation of singletons in pericentromeric regions, while pair of homologs are generally residing at euchromatic region in chromosome arms, suggesting asymmetric evolution for different members of individual whole-genome duplication (WGD)-derived gene pairs [59]. Intriguingly, the genes in pericentromeric regions where meiotic recombinations are strongly suppressed in soybean showed significantly lower rates of nonsynonymous substitution (Ka) and higher levels of expression than their homologs in chromosomal arms [59].

Tian et al. (2012) further demonstrated that the rates of local genetic recombination are negatively correlated with the densities of the nonreference LTR-RT insertions, but not with those of nonreference DNA TE insertions [60]. Distinct insertional preferences were primary factors driving purifying selection. 

## 6. Emerging Omics

As recent advances made in high-throughput DNA sequencing technologies, emerging omics, such as transcriptome, proteome, interactome, and epigenome, have been applied to soybean research. There are large numbers of next generation sequence data sets (e.g., *de novo*/resequencing of soybean cultivars and gene expression of different tissues or under different biotic or abiotic stresses) available at http://www.ncbi.nlm.nih.gov/.

### 6.1. Transcriptome

Soybean transcriptome atlases have been developed for deposit, download, or further study of transcriptional information [61]; also, the database of SoyDB (http://casp.rnet.missouri.edu/soydb/) is specifically curated for soybean transcription factors [62]. Various data sets generated using multiple tissues or different developmental stages have already been deposited. For example, we can access data generated from soybean subjected to *Pseudomonas syringae* infection [63]. Thirteen and eleven differentially expressed microsomal proteins were identified from two distinct cadmium-accumulating soybean cultivars, respectively [64].

Soybean Knowledge Base (SoyKB) is a comprehensive all-inclusive web resource for soybean research. SoyKB is designed to handle the storage and integration of the gene, genomics, EST, microarray, transcriptomics, proteomics, metabolomics, pathway, and phenotype data [65]. Other famous soybean or leguminous resources are also available, that is, Phytozome, http://www.phytozome.net/ [66]; SoyBase, http://soybase.org/ [55, 67]; Soy-TFKB (Soybean Transcription Factor Knowledge Base), http://www.igece.org/Soybean_TF/; SGMD (The Soybean Genomics and Microarray Database), http://bioinformatics.towson.edu/SGMD/ [68]; LegumeTFDB, http://legumetfdb.psc.riken.jp/index.pl [69].

### 6.2. Interactome

To understand a basic or crucial role of a given gene product in gene regulation or signal transduction, protein-protein interaction study is fundamental. The widely used systems are yeast two hybrid (Y2H), biomolecular fluorescence complementation (BiFC), affinity pull-down coupled with mass spectrometry (AP-MS), and blue native PAGE: structural analysis of protein crystals [70]. Among many systems available, one system can be used for detection and the others can serve for the verification of the putative interactions obtained. Interactome historically began with the literature survey [70]. Interactome map of *A. thaliana* was experimentally constructed via intensive screening, yielding a total of 6200 high-confidence interactions among 2700 proteins [71, 72]. In soybean, reports of interactome related to nematode resistance and sudden death syndrome have already been published [73, 74].

### 6.3. Epigenome

An epigenome is standing for a record of the chemical changes in the DNA and histone proteins of an organism. Intriguingly, these changes might be inherited by the next generation. Changes in the epigenome can result in changes in the structure of chromatin and even the function of the genome. The epigenome is involved in regulation of gene expression, development, tissue differentiation, and suppression of transposable elements. Recently, typical research subjects include the following: histone modification; for example, Chromatin Immunoprecipitation Sequencing (ChIP-Seq) identifies genome-wide patterns of histone modifications using antibodies against the modifications; DNA methylation, for example, Whole Genome Bisulfite-Seq, Reduced Representation Bisulfite-Seq (RRBS), Methylated DNA Immunoprecipitation Sequencing (MeDIP-Seq), and Methylation-Sensitive Restriction Enzyme Sequencing (MRE-Seq) [70]. Others are related to chromatin accessibility, for example, DNase I hypersensitive sites sequencing (DNase-Seq).

In soybean, DNA methylation and histone modification are revealed to be important in response to salt or salinity stress [75, 76]. 

### 6.4. Phenome

Phenotype is a general concept describing observable biological characteristics opposite to the genotype. As analytic techniques improve, phenotypes can be observed at molecular, cellular, organismal, or even population levels. The phenome generally stands for all phenotypes of an organism or a population observed. High-quality phenotypic information is so crucial for all analyses related to gene identifications, GWAS, and functional genomic and molecular breeding.

The size of a population is dependent on research purpose, for example, GWAS analysis needs a large population of over thousand individuals [70]. The accurate quantitative contents of many physiological active metabolites, for example, *α*-linolenic acid and isoflavones, in seed or other tissue, are fundamental for all genetic or genomic analysis.

## 7. Germplasm Resource

The United States Department of Agriculture National Plant Germplasm System has a collection of over 500,000 germplasm accessions including soybean and other species. In China, recently, a platform for soybean molecular breeding based on core collections of soybean germplasm has been established [77]. 

As soybean is on the list of energy crops, worldwide demands have been increased beyond the protein, oil, and physiological compounds. However, the speed of yield increase per hectare per year is far behind that of rice and maize; soybean growing area has been shrinking shapely in China. Meanwhile, the domestic demand for soybean has increased steadily year by year, leading China to be the biggest soybean import country in the world. Chinese researchers are realizing the power of molecular breeding by design by launching several research nationwide projects in order to improve the lower efficiency of traditional breeding method for good quality and high yield.

## 8. Conclusion and Future Prospective

Relative soybean complexity made soybean genome sequencing and assembling difficult several years ago; however, as tremendous progress has been made in sequencing technology, soybean genome (1.1-gigabase) was reconsidered as a reasonable genome size. In consideration of nutritional and physiological contents as well as seeds that can be used as the platform for ectopic expression of recombinant protein, soybean has possibly been regarded as a new model crop for studying the genomic duplication, gene evolution, and functional diversification. The wild soybeans are greatly different from modern cultivars in terms of flowering gene network, resistance to salt or disease, and nutritional contents. Functional analysis of many *Arabidopsis* homologs for flowering time, resistance, and other traits related genes will lead us to understand functional and evolutional diversification at genic and genomic level. Since many QTLs for important agronomic traits are genetically mapped but not cloned yet, cloning of corresponding genes will shed light on our deeper insight into gene regulation network and specific features of soybean genome. 
